# Ghana’s preparedness to exploit the medicinal value of industrial hemp

**DOI:** 10.1186/s42238-022-00167-4

**Published:** 2022-11-03

**Authors:** Richard Quansah Amissah

**Affiliations:** Department of Biomedical Sciences, University of Guelph, 50 Stone Road East, Guelph, ON N1G 2W1 Canada

**Keywords:** Industrial hemp, Ghana, Δ^9^-Tetrahydrocannabinol, Cannabidiol

## Abstract

**Background:**

Interest in industrial hemp is increasing steadily, as can be seen by the growing number of countries that have either decriminalized industrial hemp or are contemplating its decriminalization. In line with this trend, Ghana recently decriminalized the cultivation of industrial hemp (the cannabis variety with low Δ^9^-tetrahydrocannabinol (THC) and high cannabidiol (CBD) content), resulting in the need for research into its benefits to Ghanaians. This article examines cannabis (including industrial hemp) production, facilities for industrial hemp exploitation, and the potential benefits of industrial hemp in Ghana.

**Main body:**

Indigenous cannabis strains in Ghana have high THC to CBD ratios suggesting the need for the government to purchase foreign hemp seeds, considering that the alternative will require significant research into decreasing the THC to CBD ratio of indigenous cannabis strains. Furthermore, there are several facilities within the country that could be leveraged for the production of medicinal hemp-based drugs, as well as the existence of a number of possible regulatory bodies in the country, suggesting the need for less capital. Research has also shown the potential for treatment of some medical conditions prevalent among Ghanaians using medicinal hemp-based products. These reasons suggest that the most feasible option may be for the government to invest in medicinal hemp.

**Conclusion:**

Considering the challenges associated with the development of other hemp-based products, the availability of resources in the country for exploitation of medicinal hemp, and the potential benefits of hemp-based drugs to Ghanaians, investing in medicinal hemp may be the best option for the government of Ghana.

## Background

Cannabis plants can be classified as *Cannabis sativa*, *Cannabis indica*, and *Cannabis ruderalis* (Gloss 2015) based on their morphology and chemical composition. They contain different concentrations of cannabinoids (including Δ^9^-tetrahydrocannabinol (THC) and cannabidiol (CBD)) (Stott and Guy 2004). In lay terms, cannabis can be classified as industrial hemp and drug-type cannabis, where industrial hemp has a low THC (< 0.3%) to CBD ratio, while drug-type cannabis has a high THC to CBD ratio (Adhikary et al. 2021). The hemp plant is a multi-purpose plant: the seeds can be used as food for humans and livestock (Callaway 2004), the compounds in the flowering parts can used as medicine, and the fibers in the stalk can be used to produce paper, carpet, fabrics, and insulation materials (Small et al. 2002). Hemp originated from Central Asia (Klumpers and Thacker 2019), where it was mostly cultivated for food, fiber, and medicine (Russo et al. 2008; Li 1974; Collins 2020). It arrived in Europe between 1000 and 2000 BCE and in North America in the seventeenth century (Small et al. 2002), where it was cultivated mostly for fiber (Cherney and Small 2016). Africa is considered the second largest producer of cannabis herbs and the largest supplier of cannabis resins (Leggett and Pietschmann 2008). The amount of cannabis produced in Africa is enough to meet demands on the continent and beyond (United Nations Office on Drugs and Crime 2018). Cannabis was introduced into Africa along the Mediterranean coast (Duval 2019) likely through trade with Arabs (Du Toit 1976); however, it became more salient in Africa after the second world war. Its cultivation in Africa was spurred on in the 1980s by the economic crisis (Carrier and Klantschnig 2016). Cannabis was mostly smoked in Africa, but was also used for other purposes (Duval 2019). In countries like Egypt and Tunisia, which lie in the northern African region, cannabis was cultivated between the eighteenth and nineteenth centuries to meet the high demand in the region (Duval 2019). Products like edible cannabis drugs and smokable forms of cannabis, like hashish, were also common in Northern Africa (Livet 1921). In Eastern African countries like Kenya and Ethiopia, cannabis plantations were common during the thirteenth century (Du Toit 1976). Between the 1850s and 1900s, the plant grew in several locations in central Africa including Sao Tome, the democratic republic of Congo, and Angola, where it was grown as an irrigated crop (Duval 2019). In Southern Africa, the hemp variety was used to produce fibers for fabrics in Madagascar, while in Mozambique the stem was used to produce cordage and the leaves used to make medicine (Duval 2019). It was also cultivated in Botswana as an irrigated crop in the 1880s (Duval 2019). In the 1920s, cannabis reached West Africa via the coastal areas (Akyeampong 2005) through sailors and second world war veterans who returned from the Middle and Far East and North Africa (Asuni 1964). In Ghana, a West African country, the cultivation of cannabis expanded significantly between 1960 and 1980 (Bernstein 1999). Specifically, while 0.4–0.8 hectare farms were considered to be standard for cannabis farms, several 3-ha cannabis farms were identified in the country and destroyed during this period. The prevalence of cannabis use in Ghana among the population between 15 and 64 years is 21.5% (United Nations Office on Drugs and Crime 2007), which is more than five times the world average (3.4%) (United Nations office on Drugs and Crime 2000), making Ghana the leading user of cannabis in Africa and the third in the world (United Nations Office on Drugs and Crime 2019). About 50% of the harvested cannabis is exported (Bernstein 1999), earning Ghana the reputation of being one of the leading exporters of cannabis in West Africa (United Nations Office on Drugs and Crime 2020; Observatoire Geopolitique des Drogues 1995). In Ghana, cannabis is used by individuals of all classes, including students and workers (Adu-Gyamfi and Brenya 2015; Adu-Mireku 2003), by smoking, mixing with food and local beverages, and as cannabis oil (Bernstein 1999).

In the early twentieth century, hemp cultivation decreased significantly for reasons including its replacement with other materials (Small et al. 2002) and a ban on its cultivation and use (Wodak and Owens 1996; Porter 2015; Crowder 2019; Bewley-Taylor 2014). However, due to recent evidence for the potential benefits of industrial hemp and drug-type cannabis, the ban on their use for medicinal and/or other purposes has either been reversed or is in the process of being reversed in several countries (Shi 2015). Accordingly, on March 20, 2020, Ghana decriminalized industrial hemp cultivation (Owusu 2021), resulting in an increase in interest in the benefits of hemp to the country. This article examines the state of cannabis production, the facilities available to exploit industrial hemp, and the potential benefits of medicinal hemp in Ghana. In this article, medicinal hemp refers to industrial hemp prescribed for the treatment of medical conditions.

### Cannabis laws governing cultivation, usage, and trading in Ghana

Before Ghana’s independence, cannabis possession was prohibited by the 1935 Dangerous Drugs Act, which regulated the trade, production, and use of opium and other dangerous drugs (Akyeampong 2005). Ghana was also a signatory to the 1961 Single Convention, the 1971 Convention on Psychotropic Substances, and the 1988 Convention Against Illicit Traffic in Narcotic Drugs and Psychotropic Substances (Bernstein 1999). In 1990, the Narcotics Drugs Act, 1990, was passed in Ghana to criminalize the possession or importation of narcotic substances, including cannabis (Owusu 2021) and breaking this law could lead to imprisonment for up to 10 years (Sensi Seeds 2020). However, in March 2020, the Narcotics Control Commission Act, 2020 (Act 1019) was passed, which decriminalized the cultivation of hemp (which should contain no more than 0.3% THC), for individuals who have the permission to do so (Owusu 2021). The use of cannabis for medicinal purposes is still illegal, unless one has the approval of the Ministry of Health (Sensi Seeds 2020). Ghana has therefore joined African countries like Zambia, Lesotho, Malawi, Zimbabwe, and South Africa, who have already legalized cannabis cultivation (Duval 2019).

### Cannabis strains indigenous to Ghana

While the law allows accredited companies to apply for hemp cultivation licenses, none has been granted to date (Owusu 2021). Limited available data suggests that the illegal cultivation of high quality cannabis is still ongoing (Bernstein 1999; United Nations Office on Drugs and Crime 2020; Observatoire Geopolitique des Drogues 1995). In 2017, about 4.5 tons of cannabis was seized in the country (United Nations Office on Drugs and Crime 2019). Cannabis materials (leaves and flowers) seized by the Ghana Police Service across the country were highly potent with THC and CBD contents ranging from 0.93 to 15.14% and 0.88 to 1.19%, respectively (Agyepong 2019). Moreover, after analyzing the cannabinoid content of cannabis plants obtained from illegal cannabis farms located in the Ashanti, Eastern, and Volta regions of Ghana in November 2017, March 2018, and May 2018, respectively, the same study found THC and CBD contents ranging from 4.49 to 6.88% and 1.18 to 1.33%, respectively (Agyepong 2019). This suggests that cannabis plants that may be indigenous to Ghana have high THC and low CBD contents, possibly due to the weather conditions in the country (Bruci et al. 2012; Danziger and Bernstein 2021a; Danziger and Bernstein 2021b; Punja et al. 2019; Saloner and Bernstein 2021; Campiglia 2017), and therefore cannot be considered as hemp. Since hemp cultivation has been decriminalized in the country, there is the need for the government to either invest in research to decrease the THC to CBD ratio in indigenous cannabis strains, which will require significant financial and human capital, or import industrial hemp seeds that meet their requirements.

### Benefits of medicinal hemp to Ghanaians

The hemp plant has many uses. However, considering how much capital would be required to develop fiber processing plants (Fike 2016), the challenges new textile industries might face when competing with China, the leading textile producer in the world (Small et al. 2002), and the small market size for hemp-based food products, the best and fastest way for Ghana to exploit the benefits of industrial hemp may be to invest in medicinal hemp. The reasons why investing in the extraction of cannabinoids from industrial hemp may be favorable for the country include the need for less capital to establish laboratories or facilities for this purpose, the straight-forward methods for cannabinoid extraction, and the currently existing research facilities and infrastructure in the country.

Cannabinoids have been shown to be effective in treating several diseases. However, the government should focus its potential cannabinoid research towards treatments for diseases such as epilepsy and cancers that are prevalent among Ghanaians. The prevalence of epilepsy in Ghana is about 1% and the causes range from brain infections to head trauma (Ayuurebobi et al. 2015; Kariuki et al. 2014). Among people living with epilepsy in Ghana, only 15% are receiving treatment (Ayuurebobi et al. 2015). Due to the high cost associated with treatment, it is covered by Ghana’s National Health Insurance Scheme and treatments include medications such as sodium valproate, phenobarbital, carbamazepine, and phenytoin (Klevor 2020). CBD significantly reduces motor seizures by 35% in patients with severe, intractable, childhood onset, treatment-resistant epilepsy (Devinsky et al. 2016). Epidiolex, an oral solution mostly containing CBD, is also effective for the treatment of seizures (Stockings et al. 2018; Hess et al. 2016). There is therefore sufficient evidence for the efficacy of CBD against epilepsy, and this CBD could be extracted from hemp. Another disease which continues to burden Ghanaians is cancer, specifically cancers of the breast and cervix with incidences of 16.1 and 13.7 per 100,000 people, respectively (Amoako et al. 2019). Limited preliminary evidence shows that CBD can inhibit the growth and induce the death of breast and cervical cancer-causing cells (Lukhele and Motadi 2016; Shrivastava et al. 2011). Treatments for breast cancer in Ghana includes chemotherapy, radiotherapy, and mastectomy, which patients find unpleasant and expensive (Clegg-Lamptey 2009). CBD could be a cheaper and more efficient alternative drug for such conditions, which justifies the need for government to invest in medicinal hemp research.

### Improving public perception towards medicinal hemp in Ghana

The public perception towards cannabis in Ghana is negative since, in a case study published in 2005, it was reported that in Ghana cannabis is mostly associated with prostitution, violence, criminal behavior, and madness (Bernstein 1999; Sensi Seeds 2020). There is therefore a stigma associated with cannabis. This might have impacted the willingness of Ghanaians to see the crop in a different light. However, a recent cross-sectional online survey conducted between October 22 and December 10, 2021, involving 1216 respondents from six African countries reported that while majority of Ghanaians are against recreational cannabis use, similar to participants from Nigeria and Uganda, most Ghanaians are open to it being legalized for medicinal purposes, similar to participants in all countries surveyed (Kitchen et al. 2022). Specifically, 72.6% of Ghanaian respondents reported that they would be comfortable with hospitals and pharmacies selling cannabis products to patients, 69.7% reported that cannabis as medicine should only be allowed by a doctor under certain conditions, and 47.1% agreed that using cannabis as medicine is safe and effective. To improve public perception about medicinal hemp, light should be shone on the positive aspects of the plant. Through workshops and media programs (Felson false 2019), trusted experts, such as researchers and medical practitioners, in the field of medicinal hemp science could be invited to discuss the efficacy and safety of medicinal hemp-based products. The involvement of medical practitioners is very critical to the acceptance of medicinal hemp-based drugs since, following the development of drugs, they would be in the position to prescribe or recommend them to patients. Additionally, education programs should be organized for stakeholders in the development of medicinal hemp-based drugs to increase their knowledge and willingness to treat patients with and recommend the use of these products.

### Research facilities and infrastructure in Ghana for the exploitation of medicinal hemp

To understand how Ghana can benefit from medicinal hemp, it is necessary to examine existing facilities and infrastructure that can be used for testing, extraction, and research on cannabinoids. Due to the profit potential associated with the sale of cannabinoid extracts and cannabinoid-based drugs, not only the government, but other privately owned pharmaceutical companies with research facilities and resources in the country may also want a slice of the medicinal hemp market.

Even though research in Ghana is mostly funded by the government, only a small portion of the country’s annual gross domestic product (GDP) is allocated for research. For example, in 2008, only 0.3% of the country’s GDP was allocated to Research and Development (United Nations Conference on Trade and Development 2011). In Ghana, there are several public research universities and institutions whose facilities and expertise could be leveraged in order to profit from the medicinal hemp venture. Public universities with biomedical research facilities include the University of Ghana, Kwame Nkrumah University of Science and Technology, and the University of Cape Coast. Aside the numerous laboratories available in these universities, the Noguchi Memorial Institute for Medical Research, which is a part of the College of Health Sciences, University of Ghana, may also have facilities that can be used over the short-term to extract cannabinoids, evaluate their concentrations, and test their efficacies against diseases.

### Independent regulatory bodies for medicinal hemp in Ghana

In order to promote and protect public interest with regards to medicinal hemp and its products, regulatory bodies will be essential. Regulatory bodies for medicinal hemp are needed to monitor and clarify who qualifies for the license to cultivate it, where seeds can be obtained, what it can be used for, which medical conditions can be treated with medicinal hemp, which companies are allowed to produce these products, which categories of patients can be treated with medicinal hemp, when patients can be treated with medicinal hemp, the needed tests that products will have to undergo before being made available to the public, and which institutions will carry out these tests.

A possible regulatory body, already established in the country, is the Center for Plant Medicine Research (CPMR), formerly known as the Center for Scientific Research into Plant Medicine, located at Mampong. It is an agency of the Ministry of Health of Ghana. One of the services provided by the CPMR is the testing of plant-based (herbal) medicines for safety and efficacy. It has several departments including the clinical research, pharmacology/toxicology, phytochemistry, and pharmaceutics departments. The CPMR also produces laboratory animals for various research laboratories in the country. Based on the objectives of the CPMR, it could easily be one of the regulatory bodies for cannabinoid-based products. The plant development department of the CPMR could help test industrial hemp strains, how they are affected by the climate, and which species may be ideal for the production of medicinal hemp. Furthermore, knowing that factors such as light (Danziger and Bernstein 2021a), manipulation (Danziger and Bernstein 2021b), pathogens (Punja et al. 2019), nutrients (Saloner and Bernstein 2021), and plant density (Campiglia false 2017) affect the cannabinoid content and quality of hemp, the plant development department could focus on developing methods to optimize industrial hemp cultivation in Ghana to produce medicinal hemp strains, especially since cannabis strains in Ghana have high THC concentrations (Agyepong 2019).

Following the development of cannabinoid-based products by pharmaceutical companies and research facilities, the services of research centers of the Ghana Health Service like the Navrongo Health Research Center, Dodowa Health Research Center, and the Kintampo Health Research Center, could be enlisted to assess their efficacy through the conduct of clinical trials. Regulatory bodies similar to the CPMR could be established or some departments at the CPMR could be expanded in the near future based on the success of the medicinal hemp industry in the country.

## Conclusions

By focusing on the benefits medicinal hemp can offer and by putting the necessary measures in place to regulate its cultivation and processing, industrial hemp-based drugs could be affordable and excellent alternatives to several less effective drugs. Considering the current economy of Ghana, investing in industrial hemp for the production of medicine may be the most feasible option since there are currently a number of facilities that could be used for research on medicinal hemp and for the production of hemp-based medicine. Additionally, a number of institutions already present in the country can serve as regulatory bodies for hemp-based medicine. The existence of these facilities suggest the need for less capital by the government in order to exploit the benefits of medicinal hemp.

## Data Availability

Not applicable.

## Abbreviations

CBD: Cannabidiol
CPMR: Center for Plant Medicine Research
THC: Δ^9^-Tetrahydrocannabinol
